# Proteomics: a promising tool for research on sex-related differences in dioecious plants

**DOI:** 10.3389/fpls.2015.00954

**Published:** 2015-11-04

**Authors:** Le Yang, Fangping Gong, Erhui Xiong, Wei Wang

**Affiliations:** State Key Laboratory of Wheat and Maize Crop Science, Collaborative Innovation Center of Henan Grain Crops, College of Life Science, Henan Agricultural UniversityZhengzhou, China

**Keywords:** dioecious plants, proteomics, molecular biomarkers, sex determination, proteins associated with sex, sex related differences

Dioecy is a form of sex distribution in seed plants. In dioecious plants, the male and female reproductive organs sit on different unisexual plants. Dioecy occurs in many plant families, and approximately 14,600 species in 200 families are dioecious (Ming et al., 2007); however, a limited number of dioecious plants have evolved sex chromosomes (Negrutiu et al., 2001; Vyskot and Hobza, 2004). For plants without obvious sex chromosomes, genetic sex determination may be due to a single locus or multiple loci either unlinked or tightly linked on autosomes (Divashuk et al., 2014; Razumova et al., 2015). A complex sex determination mechanism has been found in *Mercurialis annua*, in which the sex is controlled by multiple unlinked loci (Janousek and Mrackova, 2010). Recently, much research on dioecious plants has focused on sex-linked genes (e.g., Blavet et al., 2015; Harkess et al., 2015; Jia et al., 2015) and sex-related differences (e.g., Chen et al., 2011, 2013; Zhou et al., 2012; Liu et al., 2013; Xiong et al., 2013; Juvany et al., 2014; Deng et al., 2015; Juvany and Munné-Bosch, 2015).

The research on sex-related differences in dioecious plants has the potential to explore the evolutionary, developmental and molecular processes leading to sex differentiation (Diggle et al., 2011) and sex chromosome evolution (Charlesworth, 2015). Unlike animals, most dioecious plants do not exhibit discernible sexual dimorphism prior to sexual maturity. In practice, the economic values often differ between male and female plants. In practice, male plants have an advantage over females in providing edible stems (e.g., asparagus, Deng et al., 2015; Harkess et al., 2015) and fibers (e.g., hemp, Divashuk et al., 2014; Razumova et al., 2015), whereas female plants are commonly cultivated for fruits (e.g., *Myrica rubra*, Jia et al., 2015) and seeds (e.g., *Pistacia chinensis*, Xiong et al., 2013). Therefore, a reliable method for sex identification at the juvenile stage would greatly benefit breeding programs for dioecious plants.

Proteomics represents a powerful tool for protein identification and gene functional analysis. In proteomic analyses, proteins are first separated using gel-based (typically 2-DE) or gel-free approaches, followed by mass spectrometry (MS). Both gel-based approaches (e.g., 2-DE) and gel-free approaches (e.g., iTRAQ) are frequently used for proteomic analysis. The aim of most proteomics analyses is to maximize the number of polypeptides that can be resolved, particularly for comparative proteomics, which generally involves identifying minor differences between experimental and control samples. In this regard, 2-DE-based proteomic analysis is particularly suitable for the paired comparison of dioecious plants. In this paper, we take a practical look at the value and the limitations of proteomic approaches for research on sex-related differences in dioecious plants.

In dioecious plants, females often invest more in reproduction and less in growth and maintenance compared to males (Barrett, 2015). This differential investment between sexes may result in distinct growth patterns (Cepeda-Cornejo and Dirzo, 2010) and sex-biased responses to environmental stresses (Xu et al., 2007; Juvany et al., 2014). Thus, it is speculated that the differences between males and females would be displayed at the protein expression level, which is the basis of the proteomic analyses of sex determination and sex-relate differences in dioecious plants. Proteomic differences are also dependent on developmental stages and environmental conditions, so experimental design is an important component. While differential abundant protein analysis has been applied to investigate sex-related differences in dioecious plants, there are only a few studies that have used proteomic approaches (e.g., Chen et al., 2011, 2013; Xiong et al., 2013).

With respect to sex determination in dioecious plants (Figure 1), to our knowledge, Bracale et al. (1990) were the first to use 2-DE to compare the differences between male and female flowers of the dioecious plant *Asparagus officinalis*; they found that the flowers exhibit a distinct set of specific proteins, some of which differed between sexes. Golan-Goldhirsh et al. (1998) analyzed differentially accumulated proteins in the inflorescence buds of *Pistacia vera* using SDS-PAGE and immunoblotting. They found that a 32 kDa glycoprotein is related to flower development and flowering in both sexes and that a 27 kDa glycoprotein is specific to females. In two species of *Actinidia*, SDS-PAGE analysis revealed specific proteins in the leaves of male and female plants: an intense band of approximately 18 kDa was specific to males, whereas an intense band of approximately 67 kDa was specific to females (Khukhunaishvili and Dzhokhadz, 2006). Differential abundant protein analysis can provide important clues in sex determination in dioecious plants, particularly in the expression of sex-related genes. Unfortunately, the above proteins were not actually identified in these studies due to technical or other limitations.

**Figure 1:**
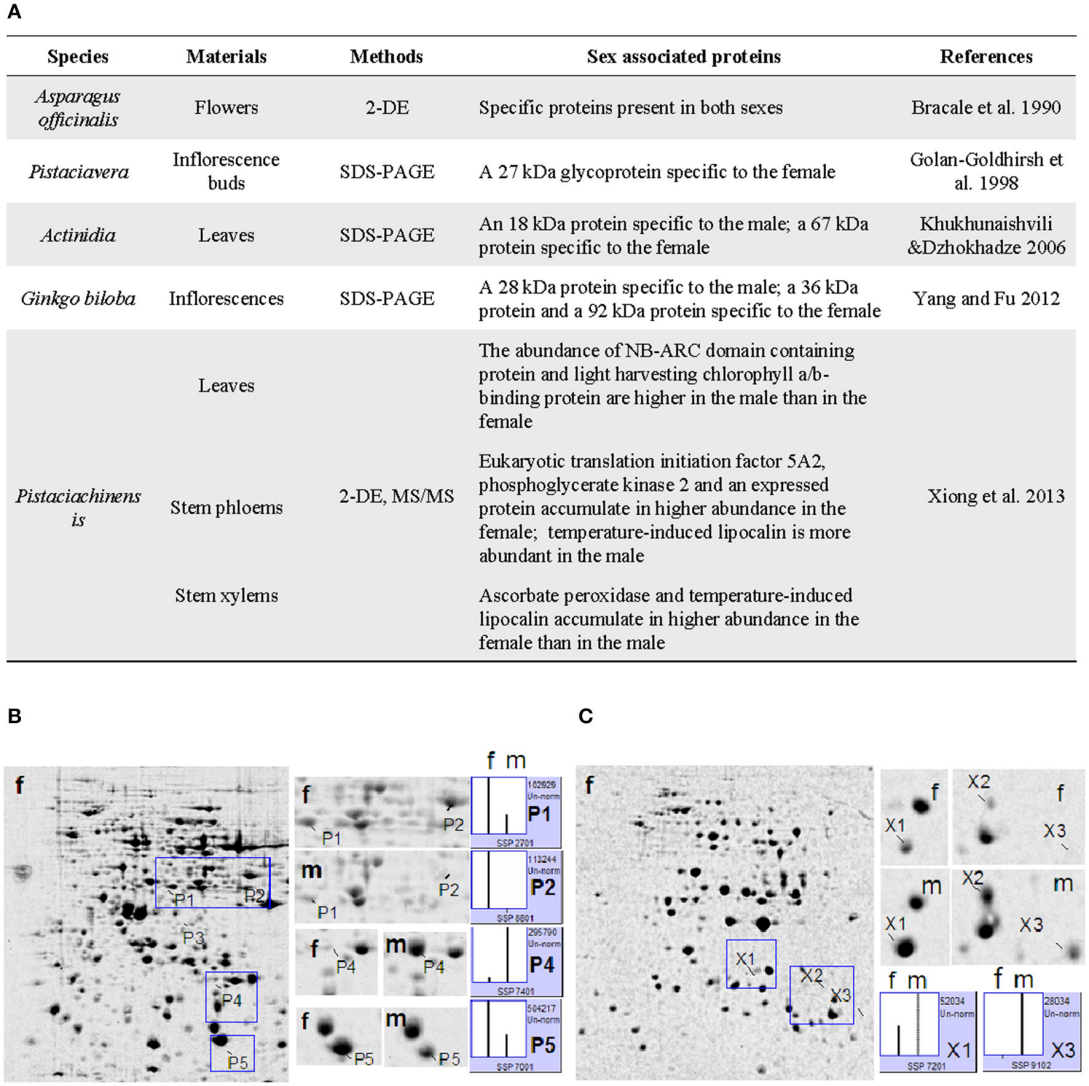
**Characterization of differentially abundant sex-related proteins in dioecious plants. (A)** Summary of several studies. **(B,C)**, 2-DE analysis of differentially abundant proteins in stem phloem **(B)** and xylem **(C)** between male and female plants in *Pistacia chinensis* (Xiong et al., 2013). A 2-DE map of female plants was used as a reference. Magnified gel regions containing differentially abundant spots were accompanied by a column configuration of relative abundance (generated via PDQUEST). The two sex-related proteins were further examined in individual plants and the results were reproducible. P1, eukaryotic translation initiation factor; P2, phosphoglycerate kinase; P5, uncharacterized protein; X1, ascorbate peroxidase; P4 and X3, temperature-induced lipocalin. P3 and X2 were not identified. f, female; m, male.

*P. chinensis* is a strict dioecious plant in the cashew family. In *P. chinensis*, male and female plants strictly maintain their respective sexual phenotypes and an approximately 1:1 sex ratio (Yu and Lu, 2011), which suggests the presence of a clear genetic basis of sex differences. This small tree or shrub is widely planted in China for biodiesel oil due to its high seed oil content (Wang and Liu, 2011). Thus, female plants of *P. chinensis* have a higher economic value than male plants. However, there are still no reliable physiological, biochemical, and molecular methods for sex identification during the long juvenile stage of this species. A similar situation exists in dioecious *M. rubra* (Chinese bayberry, Jia et al., 2015), which is an important subtropical evergreen fruit tree in southern China and Southeast Asia. Recently, we analyzed the proteomic differences associated with sex in *P. chinensis* using a 2-DE/MS-MS approach (Xiong et al., 2013). Vegetative organs (leaf and stem) of *P. chinensis*, rather than reproductive organs/tissues, were used for proteomic analysis to develop potential protein markers that can be used at the juvenile stage. Proteins from leaf, stem xylem and stem phloem were used for comparative analysis (Figure 1). Ten differential protein spots between male and female plants in *P. chinensis* were found to be reproducible, and of these ten, seven were identified via MS/MS and BLAST analysis. In particular, phosphoglycerate kinase was present in high abundance in the stem phloem in females; however, very little was detected in the males. Temperature-induced lipocalin was highly abundant in the stem xylem and stem phloem in male plants, whereas it was less abundant in female plants (Xiong et al., 2013). The abundance differences of both proteins were further confirmed in 10 individuals, sampled in autumn or in winter, indicating that they may be promising molecular marker candidates for sex determination in *P. chinensis*.

With respect to sex-related differences in dioecious plants, proteomic approach are also powerful tool when used for protein profiling differences between males and females. The Populus genus includes six dioecious species, which are all agriculturally and ecologically important trees. Abiotic stress (e.g., salinity, Mn, and Cd) is a major limiting factor for poplar growth. The completion of the *Populus trichocarpa* genome (Tuskan et al., 2006) has facilitated proteomic analysis of abiotic stress response in poplar trees. Recently, differences in the leaf proteomes of male and female *Populus cathayana* plants under excess salt (Chen et al., 2011) and Mn (Chen et al., 2013) were compared using a 2-DE/MS-MS approach. The study found that many important functional proteins are present at higher levels and there were reductions in protein degradation in males under stress conditions. Obviously, results obtained from proteomic analyses can facilitate further understandings of different management strategies of cellular activities in male and female plants, and provide gene targets for genetic manipulation of poplar tolerance to abiotic stresses.

The accurate identification and functional analysis of proteins is strongly linked to the quality and availability of the genome sequence. The majority of dioecious plants are non-model organisms with no available genome, with the exception of *P. trichocarpa*. Thus, a major limitation in proteomic analysis of sex determination in dioecious plants is the small amount of gene sequences available in public databases. The identifications of proteins from plant species with unknown genome sequences are acceptable only if MS/MS-derived peptide sequences have been used for database searching or BLAST analysis. Generally, fragment spectra should only be assigned to a peptide if the predictive value (score) is high, and such a workflow should be strictly adopted for species without a genome sequence. In the case of *P. chinensis*, six functional proteins were identified according to their high matches to homologs from *Oryza sativa, Arabidopsis thaliana, Pennisetum americanum, Ricinus communis, Solanum lycopersicum, Tamarix androssowii*, and *Vitis vinifera* (Xiong et al., 2013). Wherever possible, DNA, ESTs or protein sequences from a closely related organism should be used if the number of available sequences is low.

Despite the methodology being a relatively new, proteomics can be the method of choice to make a high-throughput discovery of sex-related differences in dioecious plants. Proteomic results can supplement and verified using physiological and molecular analyses of dioecious plants. Most importantly, the protein (particularly enzyme) information obtained via proteomic approaches provides useful clues to sex determination mechanisms and the development of molecular and biochemical detection methods for sex identification in dioecious plants. Finally, although post-genomic studies of dioecious plants is still in its infancy, continued integration of discovery-driven approaches (e.g., transcriptomics, genomics, proteomics, and metabonomics) can and will lead to unprecedented rates of information discovery in sex-linked genes and sex-related differences in dioecious plants.

## Conflict of interest statement

The authors declare that the research was conducted in the absence of any commercial or financial relationships that could be construed as a potential conflict of interest.
